# Unaffected Perceptual Thresholds for Biological and Non-Biological Form-from-Motion Perception in Autism Spectrum Conditions

**DOI:** 10.1371/journal.pone.0013491

**Published:** 2010-10-18

**Authors:** Ayse Pinar Saygin, Jennifer Cook, Sarah-Jayne Blakemore

**Affiliations:** 1 Department of Cognitive Science, University of California San Diego, La Jolla, California, United States of America; 2 Institute of Cognitive Neuroscience, University College London, London, United Kingdom; National Institute of Mental Health, United States of America

## Abstract

**Background:**

Perception of biological motion is linked to the action perception system in the human brain, abnormalities within which have been suggested to underlie impairments in social domains observed in autism spectrum conditions (ASC). However, the literature on biological motion perception in ASC is heterogeneous and it is unclear whether deficits are specific to biological motion, or might generalize to form-from-motion perception.

**Methodology and Principal Findings:**

We compared psychophysical thresholds for both biological and non-biological form-from-motion perception in adults with ASC and controls. Participants viewed point-light displays depicting a walking person (Biological Motion), a translating rectangle (Structured Object) or a translating unfamiliar shape (Unstructured Object). The figures were embedded in noise dots that moved similarly and the task was to determine direction of movement. The number of noise dots varied on each trial and perceptual thresholds were estimated adaptively. We found no evidence for an impairment in biological or non-biological object motion perception in individuals with ASC. Perceptual thresholds in the three conditions were almost identical between the ASC and control groups.

**Discussion and Conclusions:**

Impairments in biological motion and non-biological form-from-motion perception are not across the board in ASC, and are only found for some stimuli and tasks. We discuss our results in relation to other findings in the literature, the heterogeneity of which likely relates to the different tasks performed. It appears that individuals with ASC are unaffected in perceptual processing of form-from-motion, but may exhibit impairments in higher order judgments such as emotion processing. It is important to identify more specifically which processes of motion perception are impacted in ASC before a link can be made between perceptual deficits and the higher-level features of the disorder.

## Introduction

Point light displays (PLDs), which consist of a few moving points and yet evoke a clear percept of a body in motion [1] have been used for several decades to study the visual perception of biological motion [1], [2]. Despite their simplicity, PLDs are effective in representing the human body in action, as well as in conveying high-level information, such as gender, identity, intentions and emotions [2]–[5].

Autism spectrum condition (ASC) is a pervasive developmental disorder characterised by difficulties with reciprocal social interaction, in addition to unusual patterns of repetitive behaviour, and verbal and non-verbal communication problems [6]. There is currently intense interest in exploring action and body movement perception in ASC, including studies with PLDs of biological motion, which can help researchers operationalise action perception deficits based on established paradigms from vision science [2]. In particular, the perception of biological motion from PLDs has recently been linked to the brain's action perception system [7], [8], which is sometimes also referred to as the mirror neuron system [9]. Abnormalities with this system have been suggested to underlie the problems with social cognition observed in ASC [9]–[12], although there is still debate about whether this is the case [13].

A number of studies have explored whether individuals with ASC have compromised perception of biological motion, and the results are not entirely consistent. We summarize these studies in Table S1. Four of these studies [14]–[17] required participants to watch PLDs depicting either a person or an object and to describe what they see. In these studies, which have included child, adolescent, and adult populations, individuals with ASC differed from controls in their ability to recognise emotions, but not in their ability to describe actions or subjective states (such as tired or bored). These studies have suggested that individuals with ASC have an impairment in emotion recognition from PLDs.

Studies conducted with children and adolescents have tended to show atypical biological motion processing in ASC. Blake and colleagues [18] asked participants whether or not a PLD ‘moved like a person’ and found that, compared with typically developing children, 8- to 10-year-old children with ASC were impaired in this task. In a recent replication of this, Annaz and colleagues [19] showed that the performance of children with ASC did not differ from that of TD children at 4 and 5 years. However, whereas TD children showed improvement from 5 to 12 years, children with ASC showed no improvement on the task. Koldewyn and colleagues showed that adolescents with autism have decreased sensitivity to biological motion in a task that required them to determine the direction of walking of a PLD embedded in noise dots [20]. Klin and Jones reported an impairment in biological motion perception in an infant aged 15 months [21]; a follow-up study suggested that toddlers with autism may not orient to PLDs of biological motion, but instead to non-social contingencies [22].

The adult literature is more variable. Using a task that did not rely on PLDs, we recently reported that perceptual thresholds for biological and non-biological motion processing were different between adults with ASC and controls [23]. Specifically, control participants were particularly sensitive to changes in the velocity profile of biological relative to non-biological motion, whereas this increased relative sensitivity to biological motion was not found in the ASC group. In contrast, Murphy and colleagues found no impairments in adults with ASC in either accuracy or reaction times for direction detection of PLDs depicting a walking person, or a scrambled version of the same stimuli [24]. Two imaging studies [25], [26] scanned adults with ASC and Controls whilst they watched PLDs. Both studies found hypoactivation in areas typically associated with biological motion processing (such as the superior temporal sulcus and area MT/V5) in the ASC participants compared to controls, but no behavioural differences between Groups.

There are also concerns regarding how specific any impairments in biological motion perception are, given that individuals with ASC can also perform poorly in other motion perception tasks. Studies have suggested the possibility that impairments observed in ASC might be explained by problems with integrating complex perceptual information [27]. For example, using random dot kinematograms (RDKs), a number of studies have reported that participants with ASC had higher Motion Coherence Thresholds (MCTs) than controls: they required about 10% more coherent motion than did controls to report motion direction reliably [28]–[30]. It is therefore possible that individuals with ASC are less able to pool motion signals across space than controls [27], [31]. However, it should be noted that there is debate in the global motion literature with some studies finding no difference between Control and ASC groups [32] and others finding that only a subgroup of the ASC participants have motion coherence thresholds outside the normal range [31], [33]–[35]. Recently, Atkinson demonstrated a correlation between MCTs and emotion recognition from PLDs in adults with ASC, where high MCTs were associated with reduced accuracy in identifying emotions [17], and Koldewyn and colleagues observed a similar finding in adolescents [20].

**Figure 1:**
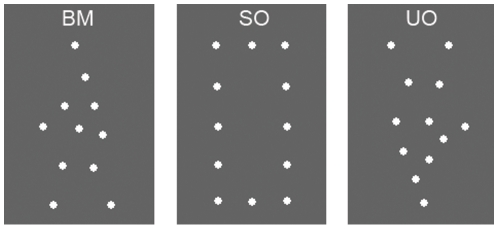
Selected frames depicting stimuli from the three conditions (BM, SO, and UO). Stimuli were point light animations composed of 12 white dots presented against a black background. In the Biological Motion (BM) condition, the stimulus was a point-light walker. In the Structured Object (SO) condition, the stimulus was a rectangle composed of point-lights. In the Unstructured Object (UO) condition, the stimulus was a single frame from the walker animation, inverted.

In the current study, we tested biological motion perception using PLDs depicting whole body movements. Biological motion not only has the dynamics of natural body movements, but also a meaningful, coherent, familiar and recognisable form. In order to tease apart these factors, as well as to assess non-biological structure-from-motion processing [36], we generated new point-light stimuli. There were three conditions: Biological Motion (BM), in which we used a point-light walker; Structured Object (SO), in which we used a translating point-light rectangle; and Unstructured Object (UO), in which we used translating set of dots comprising a meaningless, unfamiliar shape (Figure 1). Thus BM featured biological motion and a recognizable, familiar shape; SO contained non-biological form-from-motion and a familiar shape; and UO contained non-biological form-from-motion and an unfamiliar shape. In each condition, the figures were embedded in similarly moving noise dots and the task was to determine direction of movement of the figure.

A variety of different measures of biological motion processing have been employed in existing studies, ranging from *d′* as an unbiased measure of sensitivity to biological motion [18], to verbal reports [15], [16]. Here, we measured psychophysical thresholds. A Bayesian adaptive procedure was used to estimate perceptual thresholds in each condition. Given that Murphy and colleagues [24] found no difference between Control and ASC groups using a direction discrimination task similar to the one we use in the current experiment we were particularly interested to see whether our ASC group would show typical or atypical biological motion processing on our direction discrimination task.

If individuals with ASC have specific deficits in perception of biological motion perception, possibly an underlying cause of their social deficits, then we could find higher thresholds in the BM condition but not in the others. In light of some previous work (Table S1, specifically [24]), we also expected we could find no deficits in the BM condition. Non-biological form-from-motion had not been tested in previous studies. If we found increased thresholds in the non-biological motion conditions (SO and UO) in addition to the BM condition, this would reveal a more generalized deficit in form-from-motion processing, as opposed to a specific deficit for social stimuli. If we found a deficit specific to BM and SO, this could indicate that it is the processing of familiar forms that is affected in ASC. Conversely, if we found a deficit specific to UO, we could conclude that form processing is intact, or allows individuals with ASC to compensate for any deficits in from-from-motion processing. Finally, it is possible that individuals with ASC simply do not have deficits in biological or non-biological form-from-perception at this level of processing (a perceptual task), in which case thresholds would be identical to controls'.

## Methods

### Ethical Permission

Ethical permission was granted from the University College London Ethics Committee, approval No. 0858/001 and written informed consent was obtained according to Declaration of Helsinki.

### Participants

16 participants with ASC (13 males) and 20 control participants (13 males) took part (Table 1). ASC participants had a written diagnosis from a qualified clinician, which they received no more that 4 years before taking part in this experiment. ASC participants were administered the Autism Diagnostic Observation Schedule (ADOS [37]). The groups were matched for age, gender and verbal (vIQ), performance (pIQ) and full scale IQ (fsIQ) as summarized in Table S1. For the majority of participants we acquired Autistic Quotient scores [38]. Control and ASC participants had significantly different Autistic Quotient scores (ASC mean ± SD = 34.13±8.11 (N = 15); Control mean ± SD = 14.6±5.47 (N = 15); t(28) = −7.74, p<0.001).

**Table 1:** 

	ASC	NC	Group comparison
N	16	20	
Gender (M∶F)	13∶3	13∶7	
Age in years	33.75 (12.7)	37.75 (11.35)	t_(34)_ = 0.10; P = 0.33
Verbal IQ	114.00 (15.77)	114.84 (13.04) (N = 19)	t_(33)_ = 0.17; P = 0.86
Performance IQ	107.19 (14.92)	108.63 (11.76) (N = 19)	t_(33)_ = 0.32; P = 0.75
FS IQ	112.19 (16.25)	113.16 (12.35) (N = 19)	t_(33)_ = 0.20; P = 0.84

There were no significant differences between the participants in this study and those who took part in our previous study [23] in terms of age (t(65) = −0.53, p = 0.60) and full scale IQ (t(63) = 0.48, p = 0.63). The two ASC groups here and tested previously [23] did not differ in terms of ADOS total score (t (29) = −0.40, p = 0.69). 14 of the ASC participants and 10 of the Controls took part in both experiments.

### Stimuli

In all conditions, stimuli were PLDs composed of 12 white dots presented against a black background. Stimuli were presented on a CRT monitor at 1024×768 pixels resolution using Matlab (Mathworks, Natick, MA, USA) and the Psychophysics Toolbox (Brainard, 1997; Pelli, 1997). PLDs subtended approximately 4×8 degrees visual angle when viewed from 55 cm.

In the BM condition, the stimulus was a point-light walker, created by videotaping an actor and encoding the joint positions in the digitized videos [39]. In the SO condition, the stimulus was a recognisable, coherent shape (a rectangle) composed of point-lights. In the UO condition, the stimulus was an unfamiliar, less coherent shape, which was a single frame from the walker animation, inverted. Selected frames depicting all three types of stimuli are shown in Figure 1.

In the BM condition, the direction in which the point-light walker faced, right or left, was determined randomly on each trial. Like most studies on biological motion, the figure did not translate on the screen when ‘walking’ but moved as if on a treadmill. In the non-biological motion conditions (SO and UO), the shape translated at 0.5 pixels/frame either to the left or right on each trial, again randomly determined.

### Procedure

Participants were seated 55 cm from the screen with their head comfortably stabilised using a chin rest. Each trial started with a fixation cross at the centre of the screen displayed for 750 ms, after which the visual stimuli were presented for 35 frames at 60 frames/s. On each trial, the initial position of the figure was spatially jittered randomly within a 2.2° radius from the centre, in order to minimise the feasibility of a response strategy based purely on local motion information. Participants pressed one of two adjacent keys on the keyboard with their dominant hand to indicate the perceived direction of the movement (direction of walking for BM condition, direction of translation for the SO and UO conditions). If no response was given within 2000 ms from the end of the stimulus presentation, an incorrect response was registered.

Animations were presented with similarly moving ‘noise dots’ of the same shape, size and colour, a paradigm commonly used in the literature [8], [36]. To yield a psychometric measure of performance, the number of noise dots at which each participant performed at 75% accuracy was estimated using a Bayesian adaptive procedure, QUEST. In each block, a total of 60 such trials were administered and thresholds were estimated using the mean of the posterior probability density function [40].

The size of the region populated by the animations plus the noise dots was approximately 6×12 degrees of visual angle. Noise dots moved similarly to the stimuli: In the BM condition, each noise dot had the same trajectory of one of the dots in the walker. In the SO and UO conditions, the noise dots translated right or left at the same speed as the dots in the target shape. Twelve of the noise dots always translated in the direction opposite to that of the shape; since the shape was marked by 12 dots it was not possible to determine the direction of movement of the target simply from a summation of the overall movement direction in the display.

Testing sessions consisted of a practice block for each condition and three experimental blocks each of the BM, SO and UO conditions, administered in pseudo-random order across participants (e.g., First block: BM, UO, SO; Second block: UO, SO, BM; Third block: SO, BM, UO). In practice blocks, after being given instructions, participants completed 12 trials: the first 4 with no noise dots, the remaining each with a predetermined number of noise dots (5, 5, 10, 10, 25, 35, 50, 75). In the experimental blocks, there were 68 trials: the first 3 trials contained no noise dots, the next 5 trials contained a fixed number of noise dots (5, 5, 10, 30, 10), after which the QUEST procedure began with the first adaptive trial beginning at 16 noise dots. Participants could take breaks between blocks. There was also a 10 sec break after trial 45 in each block. Each experimental block lasted between 3–4 mins.

### Data analysis

The estimated number of noise dots that each participant could tolerate while performing at 75% accuracy, henceforth Noise Threshold (NT), was measured in three blocks for each condition as described above. The averages of the three NT estimates were used as dependent measures in a mixed model repeated measures ANOVA with between subjects factor Group (ASC, NC) and within subjects factor Condition (BM, SO, UO). T-tests were used to examine differences between conditions. Pearson's correlations were conducted to investigate relationships between BM, SO and UO thresholds. A Chow test was employed to examine whether the strength of these correlations differed significantly as a function of Group [41]. In addition, for the 14 ASC and 10 Control participants who took part in both the current experiment and the Cook and colleagues [23] experiment, we conducted Pearson's correlations to examine the relationship between thresholds acquired in the two studies.

## Results

The ANOVA revealed a significant main effect of condition (F(2,34) = 123.75, p<0.001). Whereas the average estimated NT was 25.72 (NC) and 25.29 (ASC) dots in the BM condition and 17.10 (NC) and 18.99 (ASC) dots in the UO condition, the SO condition was easier for both groups, with a mean NT of 70.42 (NC) and 70.10 (ASC) dots. All pairwise t-tests were significant (BM and SO: t(35) = −10.82, p<0.0001; SO and UO: t(35) = 13.11, p<0.0001; BM and UO: t(35) = 3.63, p<0.005).

**Figure 2:**
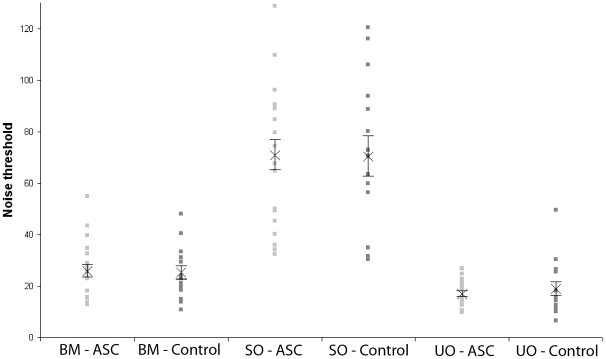
Experimental results. There was a main effect of Condition but no main effect of Group and no Group by Condition interaction. Noise threshold (NT) was higher in the Structured Object (SO) condition compared with the Biological Motion (BM) and Unstructured Object (UO) conditions, and higher in the BM condition compared with the UO condition. There was no difference between individuals with Autism Spectrum Conditions (ASC) and Normal Controls (NC).

There was no main effect of group (F(1,34)<0.0001; p = 0.99), nor was there a significant interaction between Condition and Group (F(2,34) = 0.14, p = 0.87). As shown in Figure 2, participants with ASC did not differ from NCs in any of the conditions (BM: t(34) = 0.23; p = 0.82; SO: t(34) = 0.13; p = 0.90; UO: t(34) = −0.80; p = 0.43). These effects remained insignificant when we repeated the ANOVA with covariates (IQ, age, AQ).

Across all participants BM thresholds were correlated with SO thresholds (r = 0.43; p = 0.01), as were SO and UO thresholds (r = 0.59; p<0.001). Within the control group BM and SO performance was correlated (r = 0.42; p = 0.02); but within the ASC group the correlation did not reach significance (r = 0.39; p = 0.14). Application of the Chow test showed that the relationship between BM and SO thresholds was not significantly different between the groups F(2, 31) = 0.13, p = 0.88. The SO-UO correlation was still significant within the control and ASC groups separately, and was stronger in the latter (r = −0.50, r = 0.03; p = 0.001; r = 0.68, p = 0.005), although the group difference was not significant (Chow test: F(2, 31) = 0.25, p = 0.78). BM thresholds did not correlate with age, IQ, or ADOS scores whereas SO and UO thresholds were significantly correlated with IQ (r = 0.520;p = 0.002 and r = 0.458;p = 0.006). SO and UO correlations with IQ were significant within the controls (r = 0.58; p = 0.009, and r = 0.53; p = 0.02), but weaker and short of significance in the ASC group, possibly due to the smaller sample size (r = 0.46; p = 0.08 and r = 0.44; p = 0.09). The Chow test showed no significant differences between the groups on IQ-UO correlation (F(2, 30) = 1.10, p = 0.35) or IQ-SO correlation (F(2, 30) = 0.31, p = 0.74). Across all participants, and for the ASC and Control groups separately, BO, SO and UO thresholds did not significantly correlate with thresholds for either biological or non-biological motion from Cook et al. [23] (all p<0.05).

## Discussion

There is currently intense interest in exploring the brain's action perception (or mirror neuron) system in relation to the deficits observed in autism spectrum conditions [10]–[13]. Psychophysical studies on biological motion perception allow us to test dysfunction at the perceptual levels in this system [7], [8]. However, studies that have investigated biological motion perception in ASC have produced mixed results (Table S1) and at least some aspects of biological motion processing may be preserved in ASC. Furthermore, lower sensitivity to biological motion in ASC could be explained by deficiencies in complex visual integration or by more general motion processing impairments [27]–[34]. In particular, non-biological structure from motion processing had not been tested in individuals with ASC.

In the present study, we examined psychophysical thresholds for the perception of biologically and non-biologically moving objects. Perceptual thresholds for motion detection from PLDs were measured in three conditions: Biological Motion (BM), in which we used a point-light walker; Structured Object (SO), in which we used a non-biologically moving, coherent, recognizable shape (a rectangle); and Unstructured Object (UO), in which we used a non-biologically moving, less coherent, unfamiliar shape (inverted single frame from BM condition). In all conditions the figure was embedded in noise dots that moved in the same way as the target dots and the task was to determine the direction of movement of the figure. A noise threshold was estimated in each condition adaptively [40]. We found a significant main effect of condition, broadly consistent with findings on healthy adults by Hiris [36]. Thresholds were greatest in the SO condition and lowest in the UO condition. SO featured a familiar object that has strong visual form cues (straight lines and corners), which may assist in figure-ground segregation. UO on the other hand, had no familiar form. BM lay somewhere in between in difficulty, although the raw thresholds should not be directly compared between these conditions, as the form-from-motion is depicted quite differently for BM compared to the SO and UO conditions. While in all conditions the coherence between the local motion elements defines the perceived form, in SO and UO all local elements undergo the same movement, whereas in BM the local elements undergo correlated, but non-identical movements. Consistent with this, thresholds for SO and UO conditions were strongly correlated with each other.

Our main goal here was not to look at differences between these conditions per se, but to explore if individuals with ASC differed from controls. What we found was a clear lack of a difference between groups in the perception of biological and non-biological form-from-motion – adults with ASC performed very similarly to controls for all three conditions.

Let us address the most un-interesting explanation for these results. Could it be that our participant population was too mildly affected by ASC that they are indistinguishable from controls in every task? Or is it possible that our paradigm was simply not powerful enough to detect any differences that may exist between the groups? Although such interpretations are always possible with null differences, they are unlikely to explain our data. First, we tested the same ASC population and observed significant differences from controls in previous biological motion experiments using different tasks [23]. The participants in that study largely overlapped with the present study and were not different in severity of ASC. As for the experimental design, we have used a very similar paradigm with stroke patients and observed significant differences from controls, despite the notoriously noisy nature of neuropsychological patient research [8]. We also used the same paradigm used here in a TMS experiment [42] and in a single case study with a visual agnosic patient [43]. In both studies, we found significant differences in performance with sample sizes smaller than here. Therefore the paradigm we used is sensitive enough to detect differences in performance between groups.

Our results for the BM condition are consistent with recent results from Murphy et al. [24], where participants were presented with PLDs depicting a human walker, or a spatially scrambled version of the same stimulus. As in our study, the PLD was masked with noise dots and the task was to determine the direction of movement. These authors found no differences between ASC and control participants in accuracy and reaction times in either the human walker or the scrambled walker condition. Similarly, we found no difference between ASC and control groups in direction discrimination thresholds, for PLDs depicting a walker, a non-biologically moving familiar object, or an inverted frame of a walker. Thus, we corroborate previous findings of Murphy et al. [24] and extend these results to non-biological form-from-motion. As summarized in Table S1, there are other experiments that found unaffected perceptual processing of biological motion in ASC, and it is likely that there have been other consistent observations that remain unpublished [44], [45].

Our findings are interesting in the context of prior work that has pointed to impaired global motion processing in ASC [28]–[34]. Bertone and colleagues [27] have suggested that such findings may be due to a deficiency in neuro-integrative mechanisms as manifest in impaired complex (second-order) motion processing and preserved simple (first-order) motion processing. Furthermore, Atkinson [17] has demonstrated a correlation between MCTs and biological motion processing in adults with ASC, which was also observed in adolescents recently [20]. We designed our experiment such that optimal performance would require observers to integrate the motion of the signal dots in order to perceive a coherent moving form. A deficiency in global motion processing would therefore predict lower thresholds for the ASC group compared to the NC group in all conditions. One possibility is that participants were able to use local motion cues. Although our tasks cannot be computed with no motion integration, it is possible that observers judged the direction of motion using only a subset of dots [46]. Even though the location of the stimuli was jittered from trial to trial, participants could have performed the task by identifying sub-parts of the figures (e.g., the arm of the walker, or the corner of the rectangle).

In addition, in form-from-motion perception, observers may also rely on form processing resources. A number of investigators have highlighted the role of form information in the perception of biological motion [47], [48] also supported by models of body movement perception and recent findings from neurophysiology [49], [50]. Whether observers rely on a form-based template matching strategy [47], [48] or on more dynamic representations [51], [52], we suggest that form-from-motion perception might rely on processes that are at least partially distinct from global motion processing. In support of this, Atkinson [17] reported no significant relationship between MCTs and action recognition from PLDs. Indeed, one of the stimuli used in this study was very similar to our BM condition (a PLD of an actor walking on the spot). In light of these data, it is possible that emotion recognition from PLDs relies more on global motion and form processing, whereas the recognition of the action depicted (or the detection of walking direction) may be achieved with a higher reliance on local motion cues and/or form cues. However, in a recent study, Koldewyn and colleagues [20] have found a correlation between global motion and biological motion perception in adolescents with ASC. Body movement perception likely depends on a combination of different visual cues (kinematics, featural and configural motion and form cues), and the relative contributions of these cues may differ depending on the stimuli and on task requirements [48]–[56].

Existing studies of biological motion perception have required a number of different types of judgment to be made about the stimuli, ranging from detection of the presence of a human walker in a PLD [18], to making an assessment about the perceived PLD, such as the perceived emotional state [15], [16].The type of response required may be a source of variability in this field. Both our study and Murphy et al.'s used a walker direction task (though with differences in stimuli, control conditions, and behavioural measures) and found no difference between performance in ASC and in controls [24]. The main difference between our BM condition and that utilized by Koldewyn and colleagues [20], where a significant deficit in a biological motion direction task was found in adolescents with ASC, was the masking procedure: while we used the more standard method of generating noise dots that move in the same path as the signal dots, Koldewyn and colleagues used linearly moving dots with varying degrees of coherence. So it is possible that we are tapping into slightly different segregation mechanisms. Interestingly, Koldewyn and colleagues found a correlation between biological motion perception and ADOS scores, which we did not observe in our sample. Non-biological form-from-motion was not tested in the Koldewyn study.

A related issue is whether or not participants are asked to perform a perceptual judgment. Noise thresholds from the current experiment did not correlate with noise thresholds from our previous study [23], where participants were asked to decide on the ‘naturalness’ of motion, even though most of the same participants took part in both studies. It is possible that adults with ASC struggle with biological motion tasks which require ‘higher level’ processing of the stimuli such as the attribution of emotion [14]–[17] or judgement of whether the stimulus moves in a ‘natural’ way or moves ‘like a person’ [18], [19], [23]. In contrast basic perceptual processing, such as direction discrimination may be intact [24].

To summarize, we found intact perceptual thresholds for biological and non-biological form-from-motion perception in adults with ASC. Impairments in motion and form-from-motion perception in ASC are not across the board, and are only found for some stimuli and tasks. It is important to identify more specifically which processes are impacted in ASC before a link can be made between perceptual deficits and the higher-level clinical features of the disorder.

## Supporting Information

Table S1 Literature review. Abbreviations: ADOS-G = Autism Diagnostic Observation Schedule-Generic (Lord, C., Risi, S., Lambrecht, L., Cook, E.H., Leventhal, B.L., DiLavore, P.C., Pickles, A., & Rutter, M.(2000). The Autism Diagnostic Observation Schedule-Generic: A standard measure of social and communication deficits associated with the spectrum of autism. Journal of Autism and Developmental Disorders, 30, 205–223.); AS = Asperger Syndrome; ASC = Autism Spectrum Condition; ASSQ = Autism Spectrum Screening Questionnaire (Ehlers, S., Gillberg, C., & Wing, L. (1999). A screening questionnaire for Asperger syndrome and other high-functioning autism spectrum disorders in school age children. Journal of Autism and Developmental Disorders, 29, 129–141.); BM = Biological Motion; BPVS = British Picture Vocabulary Scale; DD = developmentally delayed; CA = Chronological Age; CARS = Childhood Autism Rating Scale (Schopler, E., Reichler, R.J., & Renner, B.R.(1988). The Childhood Autism Rating Scale. Los Angeles: Western Psychological Services.); Ctrl = Control group; M = Mean age; MA = Mental Age; N = number of participants; PLD = Point Light Display, pPLD = point Point Light display (i.e., 10pPLD is a PLD composed of 10 signal dots); SD = Standard deviation of age.
(0.09 MB PDF)
