# Supplementary material for: Unaffected Perceptual Thresholds for Biological and Non-Biological Form-from-Motion Perception in Autism Spectrum Conditions
Source: PLoS One. 2010 Oct 18;5(10):e13491. doi: 10.1371/journal.pone.0013491 (PMC2956672; doi:10.1371/journal.pone.0013491)
Supplement: Table S1 — Literature review. Abbreviations: ADOS-G = Autism Diagnostic Observation Schedule-Generic (Lord, C., Risi, S., Lambrecht, L., Cook, E.H., Leventhal, B.L., DiLavore, P.C., Pickles, A., & Rutter, M.(2000). The Autism Diagnostic Observation Schedule-Generic: A standard measure of social and communication deficits associated with the spectrum of autism. Journal of Autism and Developmental Disorders, 30, 205–223.); AS = Asperger Syndrome; ASC = Autism Spectrum Condition; ASSQ = Autism Spectrum Screening Questionnaire (Ehlers, S., Gillberg, C., & Wing, L. (1999). A screening questionnaire for Asperger syndrome and other high-functioning autism spectrum disorders in school age children. Journal of Autism and Developmental Disorders, 29, 129–141.); BM = Biological Motion; BPVS = British Picture Vocabulary Scale; DD = developmentally delayed; CA = Chronological Age; CARS = Childhood Autism Rating Scale (Schopler, E., Reichler, R.J., & Renner, B.R.(1988). The Childhood Autism Rating Scale. Los Angeles: Western Psychological Services.); Ctrl = Control group; M = Mean age; MA = Mental Age; N = number of participants; PLD = Point Light Display, pPLD = point Point Light display (i.e., 10pPLD is a PLD composed of 10 signal dots); SD = Standard deviation of age. (0.09 MB PDF) [file pone.0013491.s001.pdf]

Table S1:

	Stimuli	Task	Participants	Diagnosis	Control condition?	Results	Bio. motion impairment in ASC group?
Moore, Hobson & Lee, 1997	40ms to 5000ms 5- & 10-pPLDs of walking person	Name what the dots are stuck to.	ASC: N = 17 (Range = 10-19years). Ctrls:learning disabled, matched on CA and BPVS.	ASD with impaired language processing	40ms to 5000ms 5- & 10-pPLDs moving household objects	No difference between the groups in recognition of the objects by their motions. However, ASC group had difficulty with spontaneously describing and recognising portrayals of emotion.	Yes - emotion recognition only
Blake, Turner, Smoski, Pozdol & Stone, 2003	1sec 12-pPLD of familiar activities or phase scrambled version of the same stimulus	"Does it move like a person?" - respond "yes" or "no"	ASC: N = 12 (Range = 8-10yrs). Ctrl: N = 9 (Range = 5-10 yrs). Ctrl CA matched to ASC MA	Licensed psychologist diagnosis confirmed with ADOS-G & CARS	Global-form task: Each quadrant of the screen contained short lines of randomly determined orientation, one quadrant contained a quasi-circular target. Task: Which quadrant contains the target?	The Ctrl group had significantly better d' scores for the BM task than the ASC group. Significant negative correlation between 'level' of ASC (ADOS and CARS scores) and d' score. No significant difference between the groups on the global form control task.	Yes
Herrington et al., 2007	1sec 13-pPLD walking on the spot (generated by Cutting algorithm)	Report walking direction of the PLW	ASC: N = 10, M = 27.6yrs, SD = 7.1 yrs. Ctrl: N = 10, M = 25.6yrs, SD = 4.9 yrs.	Clinical diagnosis of AS	Scrambled versions of the PLDs - each dot displaced by max. of 65 pixels in vertical dimension.	No difference between the groups on reporting walking direction. However, ASC group showed multiple hypoactivation (inc. STG and human MT+/V5)	No - but hypoactivation in bio motion areas
Hubert et al., 2007	5sec PLD of one of a) 10 actions b) 5 subjective states c) 5 emotional actions	Describe orally what is happening in the movie	ASC: N = 19, M = 21y6m, SD = 6y1m. Ctrls: N = 19, M = 24y4m, SD = 8y6m.	Clinical diagnosis of HFA or AS confirmed by ADI-R and ASSQ scale	5sec PLD of one of 5 objects	ASC group produced less accurate descriptions for the emotion PLDs only.	Yes but only on emotion labelling
Freitag et al., 2008	1.5sec 15-pPLD of walker	In scanner: assemble each PLD into a figure. Outside scanner: indicate with a button press if you see a walking person or a scrambled	ASC: N = 15, M = 17.5yrs, SD = 3.5yrs. Ctrls: N = 15 group-matched on age, sex and IQ.	Clinical diagnosis confirmed with ADI-R and ADOS-G.	Scrambled versions of PLDs (spatially scrambled & velocity profile replaced with constant velocity profile)	No difference between groups on behavioural task. ASC group showed multiple hypoactivation (inc. STS) for viewing biomotion versus scrambled compared to	No - but hypoactivation in bio motion areas

		version.				controls. Activation in intraparietal sulcus and right STS for motion (both bio and scrambled versus fixation) correlated negatively with severity of symptoms.	
Parron et al., 2008	5sec PLD of one of a) 10 actions b) 5 subjective states c) 5 emotional actions	Describe orally what is happening in the movie	ASC: N = 23, M = 11y7m, SD = 3y2m. Ctrl: N = 23, M = 12yrs, SD = 2y5m	Clinical diagnosis confirmed with ADI-R and ASSQ	5sec PLD of one of 5 objects	ASC group produced less accurate descriptions for the emotion PLDs only.	Yes but only on emotion labelling
Murphy, Brady, Fitzgerald & Troje, 2009	0.92sec 11pPLD of actor walking masked by spatially- and phase-scrambled PL dots	Report direction of walking	ASC: N = 16, M = 25.56yrs, SD = 7.67yrs. Ctrl: N = 16, M = 26.40yrs, SD = 2.85yrs, gender-matched but significantly higher non-verbal IQ than ASC group.	AS (n = 15), ASD (n = 1) diagnosed by experienced clinician	Scrambled versions of the PLDs.	ASC participants were slower overall but there were no differences between the gorups in terms of accuracy.	No
Annaz et al., 2009	1sec 13-pPLD of an actor either walking on the spot, running, throwing, kicking or star-jumping.	Report if the dots are moving like a person or not	ASC: N = 23, M = 8y1m, SD = 1y1m. Ctrl: N = 34, M = 8y3m, SD = 2y3m.	Clinical diagnosis of ASC confirmed with ADOS	Phase-scrambled versions of the PLDs.	Groups did not differ in performance at onset (4-5 years). However, performance improved with age for the TD group but not for the ASC group.	Not at younger ages but unlike Ctrls children with ASC failed to improve with age
Annaz et al., 2009	BM: PLD of actor walking on the spot embedded in scrambled PLD dots versus scrambled PLD dots. Coherent motion (CM): signal elements moving coherently versus noise elements moving randomly. Form-from-motion (FFM): signal elements arranged in a vertical rectangle surrounded by noise elements versus signal elements arranged in a horizontal rectangle surounded by noise elements (figure and noise elements moved coherently in opposite directions).	2AFC: Which panel contains the target? BM target = dots that look like a person walking. CM: target = dots that are moving in a coherent way like swimming fish. FFM: target = a rectangle that is standing up on its end.	ASC: N = 23, M = 8yr1m, SD=1y1m. Ctrl: N =34, M = 8y3m, SD = 2y3m.	Clinical diagnosis of ASC confirmed with ADOS	Static contour integration task	BM: Groups did not differ in performance at onset (4-5 years). However, performance improved with age for the Ctrl group but not for the ASC group. CM: ASC group demonstrated significantly higher MCTs than the Ctrl group. FFM: Developmental trajectories comparable but ASC group demonstrated significantly higher thresholds (more signal required) than Ctrl group.	Not at younger ages but unlike Ctrls children with ASC failed to improve with age

Koldewyn, Whitney & Rivera, 2009	Coherent motion (CM): 2sec videos of dots moving either left or right with one of six coherence levels from 0° (coherent motion) to 360°(random motion). Biological motion (BM): PLDs of actor walking masked with the CM stimuli (high coherence of the background stimulus makes PL walker easy to identify, when coherence is low PL walker difficult to identify).	CM: indicate direction of global motion. CF: indicate concentric or expanding pattern. BM: indicate direction of walking.	ASC: N = 30, M = 15.2yrs, SD = 2.64yrs,. TD: N = 32, M = 15.78yrs, SD = 2.41yrs	ADOS: social M = 8.90, SD = 2.55, comm M = 5.07, SD = 1.51, total M = 13.97, SD = 3.76	Coherent form task: Concentric glass patterns	Biological motion: ASC group exhibited significantly lower 75% thresholds. This difference is still significant if IQ is entered as a covariate. Coherent motion: ASC group exhibited significantly lower 75% thresholds. However, this difference is non-significant if IQ is entered as a covariate. Coherent Form: no group differences. Performance on bio motion and coherent motion was correlated in the ASC group but not in the control group. Performance on the coherent form task was correlated with performance on the coherent motion task for Ctrl but not ASC.	Yes
Atkinson, 2009	3 sec PL or full-light displays of either emotions or actions.	Iindicate either the emotion (5 choices) or the action (9 choices) depicted	ASC: N = 13, Range = 18-58 yrs. Ctrl: N = 16, Range = 17-54 years. Group-matched on age, full-scale IQ, verbal IQ, & performance IQ.	AS (n = 12), HFA (n = 1) confirmed by experienced clinician	Motion Coherence (MC) task: 750 dots moving in one of 5 direction randomly from frame to frame, a propotional of the dots moved coherently right or left. The task was to say if the coherently moving dots were moving left or right.	ASC group were signifcantly less accurate than the Ctrl group in classifying anger and happiness and matginally, but not significantly less accurate in classifying fear and sadness. MC accuracy significantly greater in Ctrl group than ASC group and significantly greater MCTs for the ASC group compared to the Ctrl group (borderline significantly: p = 0.054). ASC individuals with high MCT exhibited poorer emotion classification, this relationship did not hold for the Ctrl group. ASC group less accurate than Ctrl group in classifying non-instrumental actions.	Yes

Cook, Saygin, Swain & Blakemore, 2009	2sec animations of human hand moving vertically across the screen with either minimum-jerk biological motion (natural motion) constant velocity (unnatural motion) or linear combinations of these two extremes.	2AFC: Pick the less natural animation	ASC: N = 16, M = 34.1yrs, SD = 12.4 yrs. Ctrl: N = 16, M = 33.3yrs, SD = 12.2yrs.	Clinical diagnosis confirmed with ADOS. M = SD =	2sec animations of tennis ball moving vertically down the screen with either gravitational motion, constant velocity or linear combinations of these two extremes.	ASC group exhibited significantly poorer performance than the Ctrl group when required to pick the less natural hand animation but not when required to pick the less natural tennis ball animation.	Yes
Klin, Lin, Gorrindo, Ramsay & Jones, 2009	PLD of a child's game accompanied with audio	PLD and scrambled version shown on the screen simultaneously. Passive viewing task. Eyetracking data collected.	ASC: N = 21, M = 2.21yrs, SD = 0.54yrs. Ctrl: N = 39, M = 1.99yrs, SD = 0.66yrs. DD: N = 16, M = 2.02yrs, SD = 0.62yrs.	Clinical diagnosis confirmed with ADI-R and ADOS.	Inverted version of the PLD played backwards.	Whereas Ctrl and DD control groups exhibited preferential looking towards the upright PLD the ASC group did not. However, the ASD group exhibited preferential looking towards point of audio-visual synchrony.	Yes but due to focus on AV contingency (ie. not necessarily due to poor bio. motion processing)
